# Szekeres universes with homogeneous scalar fields

**DOI:** 10.1140/epjc/s10052-018-6245-7

**Published:** 2018-09-24

**Authors:** John D. Barrow, Andronikos Paliathanasis

**Affiliations:** 1 DAMTP, Centre for Mathematical Sciences, University of Cambridge, Wilberforce Rd., Cambridge, CB3 0WA UK; 2 Instituto de Ciencias Físicas y Matemáticas, Universidad Austral de Chile, Valdivia, Chile; 3 Department of Mathematics and Natural Sciences, Core Curriculum Program, Prince Mohammad Bin Fahd University, Al Khobar, 31952 Kingdom of Saudi Arabia; 4 Institute of Systems Science, Durban University of Technology, PO Box 1334, Durban, 4000 Republic of South Africa

## Abstract

We consider the existence of an “inflaton” described by an homogeneous scalar field in the Szekeres cosmological metric. The gravitational field equations are reduced to two families of solutions which describe the homogeneous Kantowski–Sachs spacetime and an inhomogeneous FLRW(-like) spacetime with spatial curvature a constant. The main differences with the original Szekeres spacetimes containing only pressure-free matter are discussed. We investigate the stability of the two families of solution by studying the critical points of the field equations. We find that there exist stable solutions which describe accelerating spatially-flat FLRW geometries.

## Introduction

The main mechanism to explain the isotropization of the observable part of the universe today from a general set of initial conditions by means of an early period of accelerated expansion, the so-called inflationary epoch, is often based on the existence of an explicit or effective scalar field dubbed the “inflaton” [1]. The scalar field temporarily dominates the expansion dynamics and drives them towards a locally isotropic and homogeneous form that leaves only very small residual anisotropies at the end of a brief inflaton-dominated period. Quantum fluctuations are also processed by the period of inflation and can manifest themselves as density and gravitational-wave inhomogeneities at late times. Consequently, pre-inflationary anisotropies could have played an important role in the evolution of the universe.

The Bianchi class of spatially homogeneous cosmologies contains several important cosmological models that have been used for the discussion of anisotropies of primordial universe and for its evolution towards the observed isotropy of the present epoch [2–6]. Detailed analysis of the Einstein field equations for Bianchi cosmologies with a cosmological constant [7], and with a changing scalar field have shown that isotropic Friedmann–Lemaître–Robertson–Walker (FLRW) attractor solutions exist for specific initial conditions when the scalar field potential has a large positive value [8]. In the case where the scalar field potential is exponential, exact solutions can be found and algebraic conditions that guarantee isotropization of ever-expanding homogeneous Bianchi universes have been derived in [9, 10] by studying the critical points of the field equations. Similar results for the Kantowski–Sachs spacetime and Bianchi types I and V had been found earlier by Burd et al. [11] although these spacetimes are not subject to the usual no-hair theorems because they have positive 3-curvature.

On the other hand, it has been found that the existence of small inhomogeneities in the spacetime does affect necessary the existence of inflation while as it has been shown by Turner et al. [12] that some homogeneous models which start from inhomogeneous models can become anisotropic in the future. However, because of the inflation that will happen in an exponentially distant time in the future, and in the present era the models to be still homogeneous up to very small ($O(10^{-5})$) metric perturbations.

An important family of inhomogeneous analytic spacetimes are the Szekeres spacetimes [13]. They belong to the class of ’Silent’ universes where information does not propagate via gravitational or sound waves. This requires the magnetic part of the Weyl tensor to be zero and the total matter source to be described by an irrotational isotropic dust fluid. An important property of the Szekeres spacetimes is that they do not admit isometries, hence these spacetimes have been characterized as “partially” locally rotational spacetimes [15]. Furthermore, it was found that the Szekeres system remains invariant with respect quantum corrections [16]. Although they possess no Killing symmetries, Szekeres universes are special in other ways because the matter distribution has a dipolar character [17]. The missing changing quadrupole ensures that there is no gravitational radiation emission from the inhomogeneously moving dust [18]. They have Newtonian analogues [19] and are the general relativistic generalisation [20–22] of the newtonian ‘pancake’ approximation introduced by Zeldovich [23].

Szekeres spacetimes are important because they have applications in many areas of gravitational physics and cosmology [24–28]. Inhomogeneous Szekeres exact solutions with a cosmological constant were derived by Barrow et al. [29], and others with a general time-dependent pressure are given in [31, 32]. The solutions of [29] have been found to be inhomogeneous generalizations of the de Sitter spacetime and were the first analytic solutions of inhomogeneous expanding inflationary spacetimes.^1^ These provide a basis for the study of non-linear inhomogeneities in inflation.

Here, we consider the Szekeres metric with a self-interacting homogeneous scalar field. The scalar field is able to describe an inflaton field and the FLRW limit exist for the resulting solutions of the field equations. For the conditions required for a FLRW limit see [33]. We know that in general the Szekeres diagonal form of the metric requires any diagonal pressure in the energy-momentum tensor to depend on time but not on space. In particular this is why exact solutions are found with dust and with dust and a cosmological constant. In the case of the homogeneous scalar field the pressure is restricted to being a function only of the time while the metric may depend on the time and space coordinates.

As in the case of the Szekeres system, with or without the cosmological constant, we find two sets of solutions which correspond to the (a) Kantowski–Sachs family and to the (b) FLRW family, of spacetimes. However, the Kantowski–Sachs family of solutions in the presence of the homogeneous scalar field turns out to be spatially homogeneous. This is not true for the second family of solutions. Specifically, the second family are inhomogeneous FLRW-like spacetimes in which the “spatial curvature” does not depend upon any variable, just as in the FLRW models. The plan of the paper is as follows.

In Sect. 2 we define our model which is a Szekeres metric with a homogeneous scalar field. The two different families of solutions of our dynamical system are presented in Sect. 3. The dynamical analysis of the critical points of the Szekeres system with the scalar field is performed in Sect. 4. Finally, in Sect. 5 we draw some conclusions.

## Szekeres system with a homogeneous scalar field

      In the context of general relativity we consider the following four-dimensional spacetime first considered by Szekeres [13]:

1 $$\begin{aligned} ds^{2}=-dt^{2}+e^{2A}dr^{2}+e^{2B}\left( dy^{2}+dz^{2}\right) , \end{aligned}$$

where $A=A\left( t,r,y,z\right)$ and $B=B\left( t,r,y,z\right)$ are to be determined by the Einstein field equations.

The energy-momentum tensor, $T_{\mu \nu }$, is assumed to be given by the expression

2 $$\begin{aligned} T_{\mu \nu }=T_{\mu \nu }^{\left( D\right) }+T_{\mu \nu }^{\left( \phi \right) }, \end{aligned}$$

where $T_{\mu \nu }^{\left( D\right) }=\rho u_{\mu }u_{\nu }$ describes a pressureless fluid source (dust) in which $u^{\mu }=\delta _{t}^{\mu }$ is the comoving 4-velocity.

We take $T_{\mu \nu }^{\left( \phi \right) }$ to be the energy-momentum tensor of a scalar field with potential, $V\left( \phi \right)$, defined as usual by

3 $$\begin{aligned} T_{\mu \nu }^{\left( \phi \right) }=\frac{1}{2}\left[ \phi _{,\mu }\phi _{,\nu }-\frac{1}{2}g_{\mu \nu }\left( \phi ^{,\mu }\phi _{,\mu }+2V(\phi )\right) \right] . \end{aligned}$$

The gravitational field equations are

4 $$\begin{aligned} G_{\mu \nu }=T_{\mu \nu }^{\left( D\right) }+T_{\mu \nu }^{\left( \phi \right) }, \end{aligned}$$

plus the separate conservation equations

5 $$\begin{aligned} T_{~\ ~~~\ ~~~~;\nu }^{\left( D\right) \mu \nu }=0,\quad T_{~\ ~~~\ ~~~~;\nu }^{\left( \phi \right) \mu \nu }=0. \end{aligned}$$

The latter dynamical system without the scalar field describes the original Szekeres system [13]. By assuming that the solution of the field equations has a FLRW limit it follows that $p_{\phi }=p_{\phi }\left( t\right)$ [33], in which $p_{\phi }=\frac{1}{3}T_{\mu \nu }^{\left( \phi \right) }\left( g^{\mu \nu }+u^{\mu }u^{\nu }\right)$.

Hence, in order for the latter to be true in the limit of the scalar field becoming a stiff perfect fluid, that is, $\phi =\phi \left( t\right)$, we consider that $\phi =\phi \left( t\right)$. Then the continuity equation $T_{~\ ~~~\ ~~~~;\nu }^{\left( \phi \right) \mu \nu }=0$ provides the differential equation

6 $$\begin{aligned} \frac{d^{2}\phi }{dt^{2}}+\left( \left( \frac{\partial A}{\partial t}\right) +2\left( \frac{\partial B}{\partial t}\right) \right) \left( \frac{d\phi }{dt}\right) +\frac{dV}{d\phi }=0, \end{aligned}$$

from which it follows that

7 $$\begin{aligned} \exp \left( A\left( t,r,y,z\right) \right) =a\exp \left( F\left( r,y,z\right) -2B\left( t,r,y,z\right) \right) . \nonumber \\ \end{aligned}$$

Thus, the spacetime metric (1) is simplified to

8 $$\begin{aligned} ds^{2}= & {} -dt^{2}+a^{2}\left( t\right) e^{2F\left( r,y,z\right) -4B\left( t,r,y,z\right) }dr^{2} \nonumber \\&+\,e^{2B\left( r,y,z\right) }\left( dy^{2} +dz^{2}\right) . \end{aligned}$$

We continue with the reduction of the field equations to a set of ordinary differential equations with respect to the comoving proper time parameter *t*, and define explicitly the geometry of the spacetime.

## Families of spacetimes

In a similar way to the case without the scalar field, the solution of the field equations is given by the two particular families of solutions where (A) $\frac{\partial B}{\partial r}=0$ and (B) $\frac{\partial B}{\partial r}\ne 0$.

### Kantowski–Sachs family: $\frac{\partial B}{\partial r}=0$

In the case in which $\frac{\partial B}{\partial r}=0$, the line element reduces to the Kantowski–Sachs spacetime,

9 $$\begin{aligned} ds^{2}= & {} -dt^{2}+a^{2}\left( t\right) dr^{2} \nonumber \\&+\,\beta ^{2}\left( t\right) e^{2C\left( y,z\right) }\left( dy^{2}+dz^{2}\right) , \end{aligned}$$

where

10 $$\begin{aligned} C\left( y,z\right) =-2\ln \left( c_{1}uv+c_{2}u+c_{3}v+c_{4}\right) \end{aligned}$$

and the new complex variables $\left\{ u,v\right\}$ are defined as

11 $$\begin{aligned} y=u+v,~z=i\left( u-v\right) , \end{aligned}$$

while the constants $c_{1}\rightarrow c_{4}$ are related to the curvature, *K*,of the two-dimensional surface $\left\{ y-z\right\}$ as follows

12 $$\begin{aligned} c_{1}c_{4}-c_{2}c_{3}=K. \end{aligned}$$

Consequently, the field equations (13)–(15) are those for the Kantowski–Sachs spacetime with a scalar field. That is, the field equations (4) and (5) are reduced to the following system of ordinary differential equations

13 $$\begin{aligned}&\frac{2}{a\beta }\left( \frac{da}{dt}\right) \left( \frac{d\beta }{dt}\right) +\frac{1}{\beta ^{2}}\left( \frac{d\beta }{dt}\right) ^{2}+\left( \frac{d\phi }{dt} \right) ^{2} \nonumber \\&\quad +\,2V\left( \phi \right) +\rho _{0}a^{-1}\beta ^{-2}+\frac{K}{\beta ^{2}}=0 \end{aligned}$$

14 $$\begin{aligned}&\frac{1}{a}\left( \frac{d^{2}a}{dt^{2}}\right) +\frac{1}{\beta }\left( \frac{d^{2}\beta }{dt^{2}}\right) +\frac{1}{a\beta }\left( \frac{da}{dt}\right) \left( \frac{d\beta }{dt}\right) \nonumber \\&\quad +\,\frac{1}{2}\left( \frac{d\phi }{dt}\right) ^{2}-2V\left( \phi \right) =0 \end{aligned}$$

15 $$\begin{aligned}&-2\left( \frac{d^{2}\beta }{dt^{2}}\right) -\frac{1}{\beta ^{2}}\left( \frac{d\beta }{dt}\right) ^{2}+\frac{K}{\beta ^{2}} \nonumber \\&\quad -\frac{1}{2}\left( \frac{d\phi }{dt}\right) ^{2}+V\left( \phi \right) =0 \end{aligned}$$

plus the conservation Eq. (6).

At this point it is interesting that the inhomogeneous spacetime (8) reduces to the homogeneous Kantowski–Sachs element and not to the inhomogeneous Kantowski–Sachs(-like) as in the case without the scalar field [13, 29, 31]. Hence, we can infer that the existence of the homogeneous scalar field provides an homogeneous anisotropic universe. The latter property is not true for the second family of solutions.

There are a few analytic solutions for the field equations (13 )–(15). For instance, a solution without the dust fluid term and with zero potential is presented in [34]; while [35] gives exact solutions are presented for string cosmologies. Recall that when $K=0$, Kantowski–Sachs spacetime reduces to the Bianchi I. For the Bianchi I spacetime analytic solutions with a scalar field without a matter source are given in [36–38]. Last, the generic vacuum solution for the Kantowski–Sachs spacetime can be found in [39]

### FLRW family: $\frac{\partial B}{\partial r}\ne 0$

For the second family of solutions, the Szekeres line element reduces to

16 $$\begin{aligned} ds^{2}= & {} -dt^{2}+a^{2}\left( t\right) \left( \left( \frac{\partial C\left( r,y,z\right) }{\partial r}\right) ^{2}dr^{2} \right. \nonumber \\&\left. +\,e^{2C\left( r,y,z\right) }\left( dy^{2}+dz^{2}\right) \right) , \end{aligned}$$

where the spatial function $C\left( r,y,z\right)$ is given by

17 $$\begin{aligned} C\left( r,y,z\right)= & {} -\ln \left( \gamma _{1}\left( r\right) uv+\gamma _{2}\left( r\right) u+\gamma _{3}\left( r\right) v+\gamma _{4}\left( r\right) \right) . \nonumber \\ \end{aligned}$$

The unctions $\gamma _{1}\left( r\right) \rightarrow \gamma _{4}\left( r\right)$ are related by

18 $$\begin{aligned} \gamma _{1}\left( r\right) \gamma _{4}\left( r\right) -\gamma _{2}\left( r\right) \gamma _{4}\left( r\right) =k, \end{aligned}$$

in which *k* is a constant and not a function of *r*, as was the case without the scalar field.

Furthermore, the scale factor $a\left( t\right)$ and the scalar field $\phi \left( t\right)$ satisfy Friedmann’s equations

19 $$\begin{aligned}&-\frac{2}{a}\frac{d^{2}a}{dt^{2}}-\left( \frac{da}{dt}\right) ^{2} +ka^{-2}-\frac{1}{2}\left( \frac{d\phi }{dt}\right) ^{2} \nonumber \\&\qquad +2V\left( \phi \right) =0, \end{aligned}$$

20 $$\begin{aligned}&\qquad -\frac{3}{a^{2}}\left( \frac{da}{dt}\right) ^{2}+\frac{3}{2}ka^{-2}+\left( \frac{d\phi }{dt}\right) ^{2} +2V\left( \phi \right) +\rho =0\nonumber \\ \end{aligned}$$

and the conservation Eq. (6).

The main difference to the case without the scalar field is that the spatial curvature *k* is a constant and not a function of *r*. However, the spacetime metric (16) remains inhomogeneous as in [13, 29, 31].

There are various analytical solutions for the field equations (19), (20) with or without the dust fluid, and with zero or nonzero spatial curvature, for instance see [41–48] while some analytical solutions with application in inflation are presented in [49, 50] and references therein.

In order to understand the evolution of the Szekeres spacetime with the scalar field and study the stability of the family of solutions that we have presented, in the next Section we perform an analysis of the critical points of the Einstein field equations.

## Dynamical evolution

We now study the dynamical evolution of the system using the covariant kinematic variables of Ehlers and Ellis [14, 51, 52]. The Einstein equations for silent universes with pressure *p*, are equivalent to the following system for the density, pressure, volume expansion rate $\theta$, shear scalar $\sigma$, and scalar electric part of the Weyl tensor, ${\mathcal {E}}$:

21 $$\begin{aligned}&\frac{d\rho }{dt}+\theta \left( \rho +p\right) =0,~ \end{aligned}$$

22 $$\begin{aligned}&\frac{d\theta }{dt}+\frac{\theta ^{2}}{3}+6\sigma ^{2}+\frac{1}{2}\left( \rho +3p\right) =0, \end{aligned}$$

23 $$\begin{aligned}&\frac{d\sigma }{dt}-\sigma ^{2}+\frac{2}{3}\theta \sigma +{\mathcal {E}} =0, \end{aligned}$$

24 $$\begin{aligned}&\frac{d{\mathcal {E}}}{dt}+3{\mathcal {E}}\sigma +\theta {\mathcal {E}}+\frac{1}{2}\left( \rho +p\right) \sigma =0, \end{aligned}$$

with the constraint

25 $$\begin{aligned} \frac{\theta ^{2}}{3}-3\sigma ^{2}+\frac{^{\left( 3\right) }R}{2}=\rho , \end{aligned}$$

where $^{\left( 3\right) }R$ denotes the curvature of the three-dimensional hypersurfaces.

The total fluid still comprises a pressureless perfect fluid (dust) and a minimally homogeneous scalar field with self-interaction potential $V(\phi )$:

26 $$\begin{aligned} \rho= & {} \rho _{D}+\rho _{\phi }=\rho _{D}+\left( \frac{1}{2}{\dot{\phi }}^{2}+V\left( \phi \right) \right) , \end{aligned}$$

27 $$\begin{aligned} p= & {} p_{\phi }=\frac{1}{2}{\dot{\phi }}^{2}-V\left( \phi \right) . \end{aligned}$$

Since the two fluids are not interacting it follows from equation (21) that

28 $$\begin{aligned}&\frac{d\rho _{D}}{dt}+\theta \rho _{D}=0, \end{aligned}$$

29 $$\begin{aligned}&\frac{d^{2}\phi }{dt^{2}}+\theta \frac{d\phi }{dt}+V\left( \phi \right) _{,\phi }=0. \end{aligned}$$

The field Eqs. (22)–(25) become

30 $$\begin{aligned}&\frac{d\theta }{dt}+\frac{\theta ^{2}}{3}+6\sigma ^{2}+\frac{1}{2}\rho _{D}+\left( \left( \frac{d\phi }{dt}\right) ^{2}-V\left( \phi \right) \right) =0, \end{aligned}$$

31 $$\begin{aligned}&\frac{d\sigma }{dt}-\sigma ^{2}+\frac{2}{3}\theta \sigma +{\mathcal {E}} =0, \end{aligned}$$

32 $$\begin{aligned}&\frac{d{\mathcal {E}}}{dt}+3{\mathcal {E}}\sigma +\theta {\mathcal {E}}+\frac{1}{2}\left( \rho _{D}+\left( \frac{d\phi }{dt}\right) ^{2}\right) \sigma =0 \end{aligned}$$

and

33 $$\begin{aligned} \frac{\theta ^{2}}{3}-3\sigma ^{2}+\frac{^{\left( 3\right) }R}{2}=\rho _{D}+\frac{1}{2}\left( \frac{d\phi }{dt}\right) ^{2}+V\left( \phi \right) . \end{aligned}$$

In order to proceed with the study of the critical points we define the new dimensionless variables scaled by appropriate powers of the volume Hubble expansion rate, $\theta :$

34 $$\begin{aligned} \Omega _{D}= & {} 3\frac{\rho _{D}}{\theta ^{2}},\quad \Sigma =\frac{\sigma }{\theta },\quad \varepsilon =\frac{{\mathcal {E}}}{\theta ^{2}},\quad y\left( t\right) =\frac{\sqrt{6}}{2\theta }\left( \frac{d\phi }{dt}\right) , \nonumber \\ z= & {} \frac{3V\left( \phi \right) }{\theta ^{2}},\quad \text {and }\quad \lambda =-\frac{V\left( \phi \right) _{,\phi }}{V}. \end{aligned}$$

Moreover, we consider the new independent variable to be $N\left( t\right)$, such that $dN\left( t\right) =\theta \left( t\right) dt$, so now the field equations (28)–(32) can be rewritten as

35 $$\begin{aligned}&\frac{d\Omega _{D}}{dN}=\frac{1}{3}\Omega _{D}\left( \Omega _{D}-1+36\Sigma ^{2}+2\left( y^{2}-z\right) \right) , \end{aligned}$$

36 $$\begin{aligned}&\frac{d\Sigma }{dN}=\frac{\Sigma }{6}\left( 4y^{2}-2z-2+6\Sigma \left( 1+6\Sigma \right) +\Omega _{D}\right) -\varepsilon ,\nonumber \\ \end{aligned}$$

37 $$\begin{aligned}&\frac{d\varepsilon }{dN}=\frac{\varepsilon }{3}\left( 4y^{2}-2z-1+9\Sigma \left( 4\Sigma -1\right) +\Omega _{D}\right) \nonumber \\&\qquad \qquad -\frac{\Sigma }{6}\left( 2y^{2}+\Omega _{D}\right) , \end{aligned}$$

38 $$\begin{aligned}&\frac{dy}{dN}=\frac{1}{6}\left( 4y^{3}+\sqrt{6}\lambda z+y\left( 2z-4+36\Sigma ^{2}+\Omega _{D}\right) \right) ,\nonumber \\ \end{aligned}$$

39 $$\begin{aligned}&\frac{dz}{dN}=\frac{z}{3}\left( 2\left( 2y^{2}-z+1\right) +36\Sigma ^{2}+\sqrt{6}\lambda y+\Omega _{D}\right) , \nonumber \\ \end{aligned}$$

and

40 $$\begin{aligned} \frac{d\lambda }{dN}=-\sqrt{6}\lambda ^{2}y\left( \Gamma \left( \lambda \right) -1\right) , \end{aligned}$$

where $$\Gamma \left( \lambda \right) =\frac{V_{,\phi \phi }V}{V_{,\phi }^{2}}$$ [53].

We assume that the scalar field potential is purely exponential, $V\left( \phi \right) =V_{0}e^{-\sigma \phi }$, so that $\lambda =\sigma$, and the resulting dynamical system is reduced from a six-dimensional to a five-dimension system comprising the differential equations (35 )–(39).

The exponential potential captures a very wide range of slow roll potentials, including power-law inflation and no inflation (for steep exponential) and de Sitter inflation when the exponent is zero. It does not possess a minimum, where non-inflationary oscillatory behaviour will occur but no exact solution will be possible. The exponential potential allows exact solutions in the homogeneous and isotropic case and so is a strong candidate for exact solutions in this inhomogeneous situation. It is also conformally related to important higher-order gravity theories with quadratic lagrangians.

The algebraic equation (33), becomes

41 $$\begin{aligned} \Omega _{R}=1-y^{2}-z-9\Sigma ^{2}-\Omega _{D}, \end{aligned}$$

with $\Omega _{R}=-\frac{3}{2}\frac{^{\left( 3\right) }R}{\theta ^{2}}$. Moreover, parameters *z* and $\Omega _{D}$ are positive parameters. The algebraic Eq. (41) is the one which defines the invariant sets on specific surfaces. For more details on the invariant sets of Bianchi cosmologies with a scalar field we refer the reader in [54].

Furthermore, at the critical points for the Raychaudhuri equation (30) it follows that

42 $$\begin{aligned} {\dot{\theta }}= & {} -\frac{1}{6}\theta ^{2}\left( 2+4y_{p}^{2}-2z_{p}+36\Sigma _{p}^{2}+\Omega _{p}\right) \nonumber \\= & {} -\frac{1}{\theta _{0}}\theta ^{2}, \end{aligned}$$

so that the solution for the expansion rate $\theta \left( t\right)$ is

43 $$\begin{aligned} \theta \left( t\right) =\frac{\theta _{0}}{t-t_{0}}. \end{aligned}$$

The dynamical system, (35)–(39), admits sixteen critical points, which form three different families. The first family (A) admits seven critical points and correspond to solutions of the system without the scalar field, that is, with $y\left( A\right) =z\left( A\right) =0\,$; however, one of the critical points corresponds to the case with $\Omega _{D}<0$, which means that it is unphysical. In the second family (B) there are two critical points. At these two points only the kinetic term of the scalar field contributes in the solution, that is, $z\left( B\right) =0=V(\phi )$, so they correspond to solutions with stiff $p=\rho$ perfect fluid. The remaining seven points correspond to the third family (C) of solutions in which $y\left( C\right) z\left( C\right) \ne 0$. However, given the condition $\Omega _{D}\ge 0$ on the density, only five points are physically acceptable.

**Table 1:** Critical points of family (A)

Point	$\left( {\Omega ,\Sigma ,\varepsilon }\right)$	$\left( {y,z}\right)$	Physical	$^{\left( 3\right) }{{R}}$	Spacetime	Stability
$A_{1}$	$\left( 1,0,0\right)$	$\left( 0,0\right)$	Yes	$=0$	FLRW (Spatially flat)	Unstable
$A_{2}$	$\left( 0,0,0\right)$	$\left( 0,0\right)$	Yes	$<0$	FLRW (Milne universe)	Unstable
$A_{3}$	$\left( 0,-\frac{1}{3},0\right)$	$\left( 0,0\right)$	Yes	$=0$	Bianchi I (Kasner universe)	Unstable
$A_{4}$	$\left( 0,\frac{1}{3},\frac{2}{9}\right)$	$\left( 0,0\right)$	Yes	$=0$	Bianchi I (Kasner universe)	Unstable
$A_{5}$	$\left( 0,\frac{1}{6},0\right)$	$\left( 0,0\right)$	Yes	$<0$	Kantowski–Sachs	Unstable
$A_{6}$	$\left( 0,-\frac{1}{12},\frac{1}{32}\right)$	$\left( 0,0\right)$	Yes	$<0$	Kantowski–Sachs	Unstable
$A_{7}$	$\left( -3,-\frac{1}{3},\frac{1}{6}\right)$	$\left( 0,0\right)$	No

**Table 2:** Critical points of family (B)

Point	$\left( {\Omega ,\Sigma ,\varepsilon }\right)$	$\left( {y,z}\right)$	Physical	$^{\left( 3\right) }{R}$	Spacetime	Stability
$B_{1}$	$\left( 0,\Sigma ,\Sigma \left( \frac{1}{3}+\Sigma \right) \right)$	$\left( \sqrt{1-9\Sigma ^{2}},0\right)$	Yes	$=0$	$$\begin{array}{c} \text {Bianchi (Kasner-like universe) for }\Sigma \ne 0\\ \text {FLRW (Spatially flat) for }\Sigma =0 \end{array}$$	Unstable
$B_{2}$	$\left( 0,\Sigma ,\Sigma \left( \frac{1}{3}+\Sigma \right) \right)$	$\left( -\sqrt{1-9\Sigma ^{2}},0\right)$	Yes	$=0$	$$\begin{array}{c} \text {Bianchi (Kasner-like universe) for }\Sigma \ne 0\\ \text {FLRW (Spatially flat) for }\Sigma =0 \end{array}$$	Unstable

From the values of the parameters at the critical points we can extract important information about the nature of the spacetime. As we discussed in the previous sections there are two possible solutions which belong to the Kantowski–Sachs and FLRW spacetimes. Hence, when the parameter $\Sigma$ vanishes, that is $\Sigma =0$, that is, the solution at the point has $\sigma =0$, the resulting spacetime is FLRW, where the value of the spatial curvature is calculated by the algebraic Eq. (41). Furthermore, the Kantowski–Sachs solutions with $\Sigma \ne 0$ are actually Bianchi I spacetimes (Kasner-like universes) when $\Omega _{R}=0$.

In Table 1 the critical points of the first family of points (A) are given, while the stability of the points is given. Similarly, Tables 2 and 3 contain the points in families (B) and (C) respectively. A discussion of the three families of critical points follows:

- *Family A*: These critical points correspond to those of the (original) Szekeres system (without the scalar field) and they were derived earlier in [14]. From the six physically accepted points, the solutions at the points $A_{1}$ and $A_{2}$ correspond to those of FLRW universe: point $A_{1}$ describes a dust solution, while $A_{2}$ describes the Milne universe. The solution of the field equations at the points $A_{3}$ and $A_{4}$ is described by the Kasner solutions of Bianchi type I spacetimes. Furthermore, Kantowski–Sachs geometries correspond to the solutions at points $A_{5}$ and $A_{6}$. From the study of the eigenvalues of the linearized system close to the critical points we can extract information about the stability of the points. We find that all the points of family (A) are unstable.
- *Family B*: The critical points of this family are surfaces because the parameter $\Sigma$ takes values in the interval $\frac{1}{3} \le \Sigma \le \frac{1}{3}$. The matter source at points $B_{1}$ and $B_{2}$ is that of a stiff fluid and corresponds to the kinetic term of the scalar field when $V\left( \phi \right) =0$. The parameter, $\varepsilon$, at these points depends upon $\Sigma$, as given by the expression $\varepsilon =\Sigma \left( \frac{1}{3}+\Sigma \right)$. Moreover, we calculate that $\Omega _{R}=0$, which means that for $\mathbf {\Sigma }\ne 0$ so the resulting solution is described by the Bianchi universe, and actually for $\Sigma \ne 0,-\frac{1}{3}$ the solution is Kasner-like [40], while for $\Sigma =-\frac{1}{3}$ the solution reduces to the Kasner universe. Finally, when $\Sigma =0$, the resulting solution is described by the spatially-flat FLRW universe with a stiff fluid. These two points are always unstable.
- *Family C*: The third family of critical points admits five physically acceptable solutions, where the potential term and the kinetic part of the scalar field contribute to the solution. Point $C_{1}$ describes a spatially-flat FLRW universe with $\Omega _{D}\left( C_{1}\right) =0$ and exists for values of $\lambda$ such that $\lambda ^{2}\le 6$, while the point is stable when $\lambda ^{2}<2$. At Point $C_{2}$ we have $\Omega _{D}\left( C_{2}\right) =1-\frac{3}{\lambda ^{2}}$, which means that the solution exists when $\lambda ^{2}>3$. Moreover the solution is described by a spatially-flat FLRW universe while the solution is always unstable. The solution at point $C_{3}$ is described by a FLRW geometry with non-zero spatial curvature, that is $^{\left( 3\right) }R=\frac{1}{3}\left( \frac{4}{\lambda ^{2}}-2\right)$. Next, we find that when $\lambda ^{2}>2$ the solution at the point $C_{3}$ is always stable, while $\Omega _{D}\left( C_{3}\right) =0$. Finally, the solutions at the points $C_{4}$ and $C_{5}$ can describe a spatially-flat FLRW geometry when $\lambda ^{2}=2$, a Kantowski–Sachs spacetime when $\lambda ^{2}>2$, and a Bianchi III geometry when $\lambda ^{2}<2$. The stability analysis shows that the solutions at these two critical points are always unstable. From the latter points we can infer that the existence of a scalar field gives solutions for the Szekeres system with positive spatial curvature.

**Table 3:** Critical points of family (C)

Point	$\left( {\Omega ,\Sigma ,\varepsilon }\right)$	$\left( {y,z}\right)$	Physical	$^{\left( 3\right) }{R}$	Spacetime	Stability
$C_{1}$	$\left( 0,0,0\right)$	$\left( \frac{\lambda }{\sqrt{6}},1-\frac{\lambda ^{2}}{6}\right)$	Yes	$=0$	FLRW (Spatially flat)	Stable $\lambda ^{2}<2$
$C_{2}$	$\left( 1-\frac{3}{\lambda ^{2}},0,0\right)$	$\left( \sqrt{\frac{3}{2}}\frac{1}{\lambda },\frac{3}{2}\lambda ^{2}\right)$	Yes	$=0$	FLRW (Spatially flat)	Unstable
$C_{3}$	$\left( 0,0,0\right)$	$\left( \sqrt{\frac{2}{3}}\frac{1}{\lambda },\frac{4}{3\lambda ^{2}}\right)$	Yes	$\ne 0$	FLRW (Spatial curvature $\frac{1}{3} ( \frac{4}{\lambda ^{2}}-2)$)	Stable $\lambda ^{2}>2$
$C_{4}$	$\left( 0,\frac{1}{6}-\frac{1}{2\left( 1+\lambda ^{2}\right) },\frac{\lambda ^{2}-1}{6\left( 1+\lambda ^{2}\right) ^{2}}\right)$	$\left( \frac{\sqrt{\frac{3}{2}\lambda }}{1+\lambda ^{2}},\frac{3}{2} \frac{2+\lambda ^{2}}{1+\lambda ^{2}}\right)$	Yes	$$\begin{array}{c} \ne 0, \lambda ^{2}\ne 2\\ =0, \lambda ^{2}=2 \end{array}$$	$$\begin{array}{l} \text {Kantowski-Sachs for }\lambda ^{2}>2\\ \text {Bianchi III for }\lambda ^{2}<2\\ \text {FLRW (Spatially flat) for }\lambda ^{2}=2 \end{array}$$	Unstable
$C_{5}$	$\left( 0,\frac{2-\lambda ^{2}}{3+12\lambda ^{2}},\frac{\lambda ^{2}\left( \lambda ^{2}-2\right) }{2\left( 1+4\lambda ^{2}\right) ^{2}}\right)$	$\left( \frac{3\sqrt{\frac{3}{2}\lambda }}{1+4\lambda ^{2}},\frac{9}{2}\frac{2+5\lambda ^{2}}{1+4\lambda ^{2}}\right)$	Yes	$$\begin{array}{l} \ne 0,~\lambda ^{2}\ne 2\\ =0,~\lambda ^{2}=2 \end{array}$$	$$\begin{array}{l} \text {Kantowski-Sachs for }\lambda ^{2}>2\\ \text {Bianchi III for }\lambda ^{2}<2\\ \text {FLRW (Spatially flat) for }\lambda ^{2}=2 \end{array}$$	Unstable
$C_{6}$	$\left( -3-\frac{3}{\lambda ^{2}},-\frac{1}{3},\frac{1}{6}\right)$	$\left( \sqrt{\frac{3}{2}}\frac{1}{\lambda },\frac{3}{2}\lambda ^{2}\right)$	No			
$C_{7}$	$\left( -\frac{3}{\lambda ^{2}},\frac{1}{6},0\right)$	$\left( \sqrt{\frac{3}{2}}\frac{1}{\lambda },\frac{3}{2}\lambda ^{2}\right)$	No			

At this point we want to discuss how the present analysis changes when we consider a potential $V\left( \phi \right)$ different from the exponential function. Technically in that consideration parameter $\lambda$ is not always a constant hence we have a six-dimensional system to study. Therefore new critical points can exists while the stability of critical points changes. More specifically, for every $\lambda =\lambda _{0}$, such that $\Gamma \left( \lambda _{0}\right) =1$, then the rhs of Eq. (40) vanishes and from the remain five equations we find the same critical points with that for the exponential potential on the surface where $\lambda =\lambda _{0}$. However, the new critical points which exists are those one where $y\rightarrow 0$, in order the rhs of (40) to be zero, and $z\ne 0$.

We find two possible points, with coordinates

$$\begin{aligned} {\mathbf {D}}_{1}&:\left( \Omega _{D_{1}},\Sigma _{D_{1}},\varepsilon _{D_{1} },y_{D_{1}},z_{D_{1}},\lambda _{D_{1}}\right) \\&\qquad =\left( 0,-\frac{1}{3},\frac{1}{3},0,3,0\right) \quad \text {and }\quad ^{\left( 3\right) }\mathbf {R>0,}\\ {\mathbf {D}}_{2}&:\left( \Omega _{D_{2}},\Sigma _{D_{2}},\varepsilon _{D_{2} },y_{D_{2}},z_{D_{2}},\lambda _{D_{2}}\right) \\&\qquad =\left( 0,0,0,0,1,0\right) \quad \text {and }\quad ^{\left( 3\right) }\mathbf {R=0.} \end{aligned}$$

At these two points parameter $\lambda$ vanishes which means that $V_{,\phi }\left( \phi \right) =0$. Therefore, the scalar field act as an cosmological constant. The curvature at point $D_{1}$ has a positive value, hence corresponds to the vacuum Bianchi III universe with cosmological constant [55]. On the other hand, at point $D_{2}$ holds, $\Sigma _{D_{2}}=\varepsilon _{D_{2}}=0$, while $^{\left( 3\right) }R\mathbf {=}0$ which means that it describes that this point describes the de Sitter universe.

We do not continue with the stability analysis of the critical points because it depends on the functional form of $\Gamma \left( \lambda \right)$, consequently of the scalar field potential $V\left( \phi \right)$.

## Conclusions

In this work we considered the Szekeres system for which we have assumed also the existence of a scalar field. The scalar field is assumed to be homogeneous so that the FLRW limit exists and the solutions of our dynamical system are comparable with the Szekeres–Szafron spacetimes. We found that there are two families of solutions which correspond to two different underlying spatial geometries. More specifically, the line element which describes the geometry can be that of the homogeneous Kantowski–Sachs spacetime or that of an inhomogeneous FLRW(-like) spacetime. The later FLRW-like spacetimes are found to be inhomogeneous, but their spatial curvature is not an arbitrary function of one of the spatial variables as in the Szekeres system. Furthermore we find that the constants of integration for the reduced system of the gravitational field equations are constants and not functions of one of the space variables as in the Szekeres geometries.

The second main difference with the Szekeres spacetimes is that the first family of Kantowski–Sachs spacetimes are spatially homogeneous and not inhomogeneous, that is, the first family of solutions are locally rotational symmetric spacetimes which means that they admit a four-dimensional Killing algebra, while the Szekeres spacetimes containing only dust do not admit any Killing fields.

In order to study the stability of the two different spacetimes we performed an analysis of the critical points for the gravitational field equations. In order to perform that analysis we wrote the field equations in terms of the kinematic quantities for the comoving observer, $u^{\mu }=\delta _{0}^{\mu }$, and we normalized the parameters according to the $\theta$-normalization. In order to perform our analysis we assumed the scalar field potential to be exponential. We found three different families of critical points which correspond to the (A) Szekeres system, (B) Szekeres system with stiff fluid and (C) solutions where both the kinetic and potential parts of the scalar field contribute.

The critical points of families (A) and (B) are found to be always unstable, while there are only two possible stable solutions which belong to the third family. Indeed, the possible stable solutions are points $C_{1}$ and $C_{3}$. Point $C_{1}$ can describe an accelerated universe when it is stable, while the solution at point $C_{3}$ is stable when it describes an open universe. On the other hand, we found that there are two critical points, namely $C_{4}$ and $C_{5}$ which can describe solutions with a homogeneous Bianchi III geometry.

We conclude that the existence of an “inflaton” in the Szekeres system can lead to inhomogeneous accelerated FLRW-like universes. Such an analysis is important in the pre-inflationary epoch and our solutions extend the inhomogeneous de Sitter generalizations of [29]. In a forthcoming work we will generalize this analysis to the Szekeres–Szafron system.

## Footnotes

1 Szekeres metrics in $2+1$ dimensional spacetimes were found in ref [30].
